# T Lymphocyte Autoreactivity in Inflammatory Mechanisms Regulating Atherosclerosis

**DOI:** 10.1100/2012/157534

**Published:** 2012-12-10

**Authors:** Elisabetta Profumo, Brigitta Buttari, Luciano Saso, Raffaele Capoano, Bruno Salvati, Rachele Riganò

**Affiliations:** ^1^Dipartimento di Malattie Infettive, Parassitarie ed Immunomediate, Istituto Superiore di Sanità, viale Regina Elena 299, 00161 Rome, Italy; ^2^Dipartimento di Fisiologia e Farmacologia “Vittorio Erspamer”, Sapienza Università di Roma, Piazzale Aldo Moro, 00185 Rome, Italy; ^3^Dipartimento di Scienze Chirurgiche, Policlinico Umberto I, Sapienza Università di Roma, Piazzale Aldo Moro, 00185 Rome, Italy

## Abstract

Atherosclerosis has been clearly demonstrated to be a chronic inflammatory disease of the arterial wall. Both cells of the innate and the acquired immune system, particularly monocytes and T lymphocytes, are implicated in the atherogenic process, producing different cytokines with pro- and anti-inflammatory effects. The majority of pathogenic T cells involved in atherosclerosis are of the Th1 profile, that has been correlated positively with coronary artery disease. Many studies conducted to evaluate the molecular factors responsible for the activation of T cells have demonstrated that the main antigenic targets in atherosclerosis are modified endogenous structures. These self-molecules activate autoimmune reactions mainly characterized by the production of Th1 cytokines, thus sustaining the inflammatory mechanisms involved in endothelial dysfunction and plaque development. In this paper we will summarize the different T-cell subsets involved in atherosclerosis and the best characterized autoantigens involved in cardiovascular inflammation.

## 1. Atherosclerosis and Inflammation

Atherosclerosis represents the leading cause of morbidity and mortality in industrialized countries. It was initially believed as a process caused by the passive accumulation of lipids in the vessel wall, whereas nowadays it is considered as a complex condition where multiple pathogenic factors contribute to trigger and sustain vessel wall damage [1]. Experimental studies have clearly demonstrated that atherosclerosis can be considered as a chronic inflammatory disease of the arterial wall and that inflammation plays a key role in all stages of the pathogenic process, including formation, progression, and rupture of atherosclerotic plaque [2, 3]. 

Inflammation related to atherosclerosis has been extensively studied, and a variety of inflammatory mediators positively correlate with the underlying disease load. These mediators regulate every stage of the inflammatory cascade, from endothelial activation to the adhesion of inflammatory cells and platelets, and subsequent remodelling of the internal vascular environment. In this way, inflammatory mediators affect every step of the atherosclerotic process, from emergence of the fatty streak—the first identifiable lesion of atherosclerosis—to subsequent vascular dysfunction and vessel occlusion [4]. 

Among inflammatory molecular mediators, a key role is played by cytokines and chemokines, which modulate all aspects of vascular inflammation by altering the proliferation, differentiation, and function of vascular and immune cells. These regulatory mediators and their receptors have been demonstrated in atheromatous tissue where they modulate plaque morphology and stabilization. In early lesions, chemokines and adhesion molecules expressed by endothelial cells orchestrate adhesive interactions of circulating immune cells, in particular monocytes and T cells, with the arterial wall and their subsequent extravasation [5]. Exhibiting a high degree of specialization and cooperation, different chemokines mediate distinct steps during the atherogenic recruitment of inflammatory cells [6]. 

Clinical and experimental data have demonstrated that cells of both the innate (monocyte-derived macrophages and dendritic cells) and the acquired immune system (T and B lymphocytes) are implicated in the atherogenic process, producing a wide array of cytokines that can exert both pro- and anti-inflammatory effects [7–9]. Monocytes, migrated from the circulation into the intima of the arterial wall differentiate into macrophages and dendritic cells (DCs), take up modified lipoproteins, and transform into foam cells. Monocyte-derived macrophages are abundantly present in every stage of the disease process. Their pivotal role in atherogenesis has been demonstrated by the attenuation of lesion formation in monocyte-deficient ApoE knockout mice and LDL receptor knockout mice [10, 11]. DCs are the immune cells that internalize, process, and present antigen, leading to activation or suppression of T cells [12]. As key modulators of immune responses, they are likely to play a crucial role in directing innate or adaptive immunity against candidate antigens involved in atherosclerotic disease [13]. Several studies have implicated the role of these cells in progression and destabilization of the atherosclerotic plaque [14–16]. If the acute inflammatory response does not resolve and macrophages and T cells continue to accumulate in the intima, a fibrotic repair process activates, leading to the formation of an atherosclerotic lesion which consists of a lipid core covered by a fibrotic cap on the luminal surface [17]. This process is driven by the inflammatory cells that secrete cytokines and growth factors and stimulate smooth muscle cells to migrate to the intima, where they proliferate and produce extracellular matrix proteins [5]. Integrity of the fibrous cap depends on the balance between the smooth muscle cell proliferation and migration, and matrix metalloproteinase-induced degradation of the extracellular matrix. In advanced lesions, metalloproteinases secreted by activated macrophages degrade the connective tissue in the fibrous cap, thus causing plaque rupture.

It is becoming clear that plaque progression to an unstable phenotype, susceptible to rupture and responsible for thromboembolic events, depends on the cell subtypes and on the specific molecular mediators accumulating within the plaque. A number of studies have been performed aimed to elucidate this issue, especially focusing on molecular and cellular mediators stimulating the thinning of the fibrous cap and its subsequent rupture. Although initial research focused more on pro-inflammatory cytokines in relation to disease progression, it is the imbalance between pro- and anti-inflammatory cytokines produced by different types of inflammatory and immune cells that is emerging to be pivotal in determining the structure of the fibrous cap, thereby influencing plaque stability [4, 9].

Considering that pro- and anti-inflammatory balance is a major determinant of disease progression, many studies have been performed to evaluate whether circulating levels of cytokines are associated to atherosclerotic disease. Several studies have demonstrated that serum levels of pro-inflammatory T helper (Th) 1-related cytokines positively correlated with the severity of atherosclerotic disease [18–25]. In particular, high circulating levels of tumour necrosis factor (TNF)-*α* and interleukin (IL)-6 were predictors of incident coronary and cardiovascular events, whereas high levels of the anti-inflammatory cytokine IL-10 were associated with a significantly improved outcome of patients with acute coronary syndromes [18–20]. Moreover, prospective epidemiological studies found increased risk of incident or recurrent coronary heart disease associated with increased baseline levels of monocyte chemoattractant protein- (MCP-) 1 [23]. Evaluation of IL-18 and IL-2 serum levels in carotid atherosclerosis revealed an association of these cytokines with intima media thickness [21, 22]. In line with these studies, we found that intracellular levels of the pro-inflammatory cytokines TNF-*α*, interferon (IFN)-*γ*, IL-1*β*, IL-6, and IL-8, in the peripheral blood lymphocytes and monocytes from patients with carotid atherosclerosis were associated with the outcome of contralateral disease after carotid endarterectomy (CEA). We suggested that increased levels of these pro-inflammatory cytokines might be a warning signal indicating progressive carotid atherosclerosis [24]. Furthermore, intracellular pro-inflammatory cytokine expression was significantly higher in patients undergoing CEA (patients with stenosis ≥70%, previous stroke or amaurosis fugax) than in patients with a stable plaque without indication for CEA, suggesting the presence of an association between increased levels of pro-inflammatory mediators and clinical or ultrasound indications for surgery [25].

All these findings support the concept that detection of cellular activation in the peripheral blood is a good indicator of inflammatory mechanisms taking place within the atherosclerotic plaque.

## 2. T Lymphocyte Response in Atherosclerosis

There is now ample evidence from experimental studies that the adaptive immune system affects the development of atherosclerosis. Deficiency in both T and B cells determines a reduction in atherosclerotic lesion development, as shown in apolipoprotein (Apo)E-deficient mice [26–29]. In accordance with these results, transfer of CD4+ T cells aggravates atherosclerosis in immunodeficient ApoE knockout mice, indicating a proatherogenic role for T cells [30]. Following the demonstration of a pathogenic role for T cells in atherosclerosis, several groups have been involved in the characterization of the pathogenic T-cell subsets. It is now clear that most of the T cells in atherosclerotic plaques are CD4+ T cells expressing *αβ*-TCR, which interacts with MHC class II molecules [31]. Furthermore the majority of pathogenic T cells in atherosclerosis are of the Th1 profile, producing pro-inflammatory mediators such as IFN-*γ* and activating macrophages [32, 33]. Th1-driven responses are detrimental to the atherosclerotic process. IFN-*γ* inhibits the synthesis of collagen by the vascular smooth muscle cells, damaging the protective thick fibrous cap of the plaque. It also activates monocytes/macrophages and dendritic cells, leading to the perpetuation of the pathogenic Th1 response [31]. Deficiency in IFN-*γ* or in its receptor significantly reduces lesion development and enhances plaque collagen content, whereas exogenous administration of IFN-*γ* enhances lesion development [34, 35]. More recently, it has been shown that postnatal blocking of IFN-*γ* function in ApoE knockout mice prevents the progression of established plaques that remodel toward a more stable and less inflammatory phenotype [36]. In a study aimed to assess the molecular mediators associated with atherosclerotic plaque phenotype by the use of multiplex technology, we observed that complicated plaques secreted higher levels of the interferon-*γ*-induced protein- (IP-) 10 and lower levels of IL-5 compared to uncomplicated ones [37]. IP-10 is a chemoattractant protein which promotes adhesion of T cells to endothelial cells and is associated with the Th1 response, whereas IL-5 is a Th2 cytokine involved in antibody production [38, 39]. Therefore, our data confirmed a preferential Th1/pro-inflammatory activation associated with atherosclerotic lesion progression. 

Th2 cells are rarely detected within the atherosclerotic lesions. They secrete IL-4, IL-5, IL-9, and IL-13 and provide help for antibody production by B cells. As these cells downregulate IFN-*γ* production, Th2-biased responses were proposed to antagonize proatherogenic Th1 effects, thus conferring atheroprotection. However, the role of the Th2 pathway in the development of atherosclerosis remains controversial and depends on the stage and site of the lesion, as well as on the experimental model used [32]. Studies of animal models have suggested that both Th1 and Th2 responses play roles throughout the development of atherosclerosis, Th1 activation being predominant during the initiation of lesion formation with a switch toward a proatherogenic Th2 response in the chronic phase of plaque development [40]. Furthermore, in mouse models that are relatively resistant to atherosclerosis, a Th2 bias has been shown to protect against early fatty streak development [41]. In contrast, in LDL receptor knockout mice, an experimental model more permissive to atherosclerotic disease development, deficiency in IL-4, the prototypical Th2-related cytokine, was associated with a decrease in atherosclerotic lesion formation, suggesting a potentially proatherogenic role of this cytokine [42]. 

Another T helper subset involved in inflammation is represented by Th17 lymphocytes, which are a new lineage distinct from Th1 and Th2 [43]. Th17 cells produce IL-17 and are involved in the development of a wide range of autoimmune diseases, such as rheumatoid arthritis, multiple sclerosis, psoriasis, and inflammatory bowel disease [43]. Both protective and pathogenic effects have been described for IL-17 in distinct autoimmune diseases [44, 45]. The precise role of IL-17 in atherosclerosis is still controversial because of contradictory publications on the subject [46]. Many studies provide evidence that IL-17 is proatherogenic [47–51]. A proof of IL-17 proatherogenic effect is the observation that CD4+ T cells isolated from atherosclerotic coronary vessels express both IL-17 and IFN-*γ* [52]. Furthermore, increased frequencies of circulating Th17 cells and Th17-associated cytokines correlated to the severity and progression of carotid artery plaques [53]. Other studies of experimental models and humans report an anti-atherogenic role for IL-17. Promotion of Th17 responses by genetic inactivation of SOCS3 in T cells reduced atherosclerosis in LDL receptor knockout mice [54]. Interestingly, higher IL-17 expression and STAT3 phosphorylation in human carotid plaques were associated with a more stable phenotype [54]. 

Analysis of T-cell subsets in the peripheral blood of patients with coronary artery disease revealed the expansion of an unusual subset of T cells named CD4+CD28null characterised by the lack of the costimulatory molecule CD28. These cells, expanded in patients with unstable angina and infrequent in those with stable angina, produce high levels of IFN-*γ* and TNF-*α*. This finding suggests that CD4+CD28null T cells may be involved in lesion progression and instability [55–58]. 

CD4+ T cells also include a subset with anti-atherosclerotic properties, the regulatory T cells (Tregs). They have anti-inflammatory, immunoregulatory, and suppressive properties and are involved in the modulation of adaptive immune responses, being able to suppress effector CD4+ and CD8+ T cells and to induce tolerance [59, 60]. Different subpopulations of regulatory T cells are known. The intracellular transcription factor Foxp3, which acts as a silencer of cytokine gene promoters and programs the development and function of Tregs, is a marker of natural Tregs (nTreg), CD4+CD25+ T lymphocytes generated through selection in the thymus [61, 62]. Other subpopulations of regulatory T cells are inducible Tregs (iTregs), generated from the conversion of naïve T cells in peripheral lymphoid tissues, and Tr1 cells, an IL-10-induced subset of CD4+ T cells [59]. A role for natural Tregs in experimental murine atherosclerosis was initially reported by Ait-Oufella and colleagues. They demonstrated that depletion of peripheral Tregs increased atherosclerotic lesion size and vulnerability in ApoE knockout mice [63]. This finding was further strengthened by the study of Mor et al., who reported reduced Treg numbers and compromised suppressive function in atherosclerotic ApoE knockout mice compared to control mice [64]. Transfer of Tregs to ApoE knockout mice resulted in reduced atherosclerotic lesion formation and reduced plaque rupture susceptibility. The protective role of Treg in atherosclerosis was confirmed in humans, in patients with acute coronary syndrome who showed significantly reduced Treg levels and suppressive functions as compared to patients with stable angina and to normal coronary artery subjects [65–67]. Very recently George et al. confirmed that patients with clinically stable atherosclerotic plaques had higher levels of Tregs and of the cytokine IL-10 than vulnerable patients presenting with recurrent myocardial infarctions, despite a similar cardiovascular risk profile and a comparable extent of coronary artery disease [33]. Tregs exert immunoregulatory and suppressive functions through the production of the anti-inflammatory cytokines transforming growth factor (TGF)-*β* and IL-10 [59]. The protective role of these cytokines in atherosclerotic lesion formation and stability had been previously reported in *in vitro* and *in vivo* studies of animals and humans [63, 68–73]. 

Another T-cell type named TCR*γδ*+ CD4− CD8− (double negative T cells) that can suppress proliferation and cytokine production of highly activated effector T cells may also have antiatherogenic properties which need to be better addressed [58, 74]. 

Regarding CD8+ T cells, deficiency of these cells in ApoE knockout mice showed no major influence on atherogenesis [75]. In contrast, the finding that CD8+ T cell recruitment into lesions after stimulation with a CD8+ T cell stimulatory agonist antibody [76] caused a parallel increase of lesion size suggests a pathogenic role for these cells [58].

In order to understand the role of different immune cells in cardiovascular disease onset and progression, it is crucial to consider that the various cell types involved in atheroma formation, including T cells, macrophages, dendritic cells, and vascular endothelial cells, can reciprocally modulate each others' functions. This aspect must be taken into account in the efforts to develop novel strategies aimed to control the inflammatory processes involved in atherogenesis.

## 3. Antigens Inducing T Lymphocyte Activation in Atherosclerosis

A number of data from humans and mice show oligoclonal expansion of T cells within atherosclerotic lesions owing to the preferential expression of a limited number of TCR-variable gene segments [77, 78]. This suggests that a limited set of candidate antigens mediates the specific T-cell proliferation. Many studies have been conducted to evaluate the molecular factors responsible for the activation of the adaptive immune system which affects the development of atherosclerosis. Some of these studies indicate infectious agents as possible inducers of immune system activation [79, 80]. It has been reported that the occurrence of respiratory infections increases the risk of acute coronary syndrome and is associated to myocardial infarction in humans [81, 82]. Furthermore, the chronic infectious agents *Chlamydia pneumoniae* and *Porphyromonas gingivalis* have been linked to atherosclerosis and to increased risk of acute coronary events [83–85]. However, the direct role of infectious agents in cardiovascular diseases is controversial and has not been supported by strong evidence [86].

Many experimental and clinical data suggest that the main antigenic targets in atherosclerosis are modified endogenous structures [87]. Atherosclerotic plaques express autoantigens that are targeted by both IgM and IgG. It is likely that these autoimmune responses initially have a beneficial effect facilitating the removal of potentially harmful antigens [7]. However, studies performed on hypercholesterolaemic mice deficient in different components of innate and adaptive immunity uniformly indicate that the net effect of immune activation is proatherogenic [7, 88] and that atherosclerosis, at least to some extent, should be regarded as an autoimmune disease. 

Oxidative stress, increasingly reported in patients with atherosclerosis, is the major event causing protein structural modifications. The best characterized modified autoantigens in atherosclerosis are the oxidized low-density lipoproteins (LDLs). The oxidation process is associated with major structural modifications of LDLs and determine the formation of new antigenic epitopes which can be presented by dendritic cells and give rise to clonal expansion of oxidized LDL-specific T cells [87]. About 10% of all T cells present in human atherosclerotic plaques specifically recognize oxidized LDLs [78], and these cells are also present in the circulation [89]. Pilot studies in animal models have provided promising results for the development of vaccines based on oxidized LDL antigens [87]. 

A second category of autoantigens that have been implicated in atherosclerosis are the stress-induced heat shock proteins (HSPs) [90]. HSPs act as molecular chaperons facilitating refolding of denatured proteins in stressed cells and increase in response to many environmental stresses, including oxidative stress [91]. Under stress conditions, HSPs are expressed not only within cells, but also on the cell surface and can be released into the intercellular space. In atherosclerotic lesions, human HSPs appear to stimulate an immune response leading to the development and progression of atherosclerosis [92–94]. Antibody levels against HSP60/65 are increased in subjects with cardiovascular disease and are associated with disease outcome. Different vaccination protocols based on these proteins protect against progression of early atherosclerosis by inducing Treg activation [95, 96]. Recently, we suggested that human HSP90 is a possible autoantigen involved in the pathogenesis of carotid atherosclerosis [97]. This self-molecule is overexpressed in plaque and serum from patients with carotid atherosclerosis and induces immune responses in these patients. In particular, we detected within the atherosclerotic plaque HSP90-specific T lymphocytes with a predominant Th1, pro-inflammatory profile. We suggested a role for HSP90 in sustaining the inflammatory mechanisms responsible for the thrombogenicity of the atherosclerotic lesion [97]. 

Other self-molecules suggested as target of immune responses when modified by oxidative stress are the plasma protein beta2-glycoprotein I (*β*2-GPI) and haemoglobin (Hb) [98]. *β*2-GPI is the most common target for antiphospholipid antibodies, which play a key role in thrombotic events and in the incidence of accelerated atherosclerosis in patients with the antiphospholipid syndrome and with systemic lupus erythematosus [86, 99, 100]. Previous studies of animal models demonstrated that the transfer of lymphocytes obtained from *β*2-GPI-immunized mice was associated with the presence of larger fatty streaks within the recipients compared with animals that received lymphocytes from control mice. This result suggested that *β*2-GPI is a target autoantigen in atherosclerosis and T cells specific for *β*2-GPI have a pathogenetic role [101]. In line with this result, we recently found that *β*2-GPI is a target antigen of Th1 cellular immune response in patients with carotid atherosclerosis [102]. We had previously demonstrated that *β*2-GPI undergoes major structural and functional changes upon exposure to oxidative stress rendering this self-molecule able to activate immature monocyte-derived DCs. DCs, stimulated with the oxidized form of human *β*2-GPI acquire a mature phenotype, produce cytokines that support T lymphocyte activation and favour a Th1-type response by allogeneic naïve T lymphocytes [103, 104]. 

Concerning the possible role of Hb as a candidate autoantigen in atherosclerosis, in a recent paper we demonstrated the presence within human atherosclerotic plaques of T lymphocytes specific for oxidized Hb and producing high levels of the pro-inflammatory Th1 cytokines IFN-*γ* and TNF-*α* [105]. Complicated plaques are characterized by repetitive haemorrhage events and hemolysis, accompanied with the release of large amounts of Hb into the atherosclerotic lesions [106]. In these conditions, the capacity of Hb scavenging mechanisms is saturated. The prooxidant microenvironment within the lesion may predispose free Hb to oxidative modifications, determining the activation of specific pro-inflammatory T lymphocytes. These observations indicate that this protein, in particular conditions, can activate immune cells, thus promoting the pathogenetic mechanisms involved in the progression of cardiovascular diseases [98]. 

**Figure 1:**
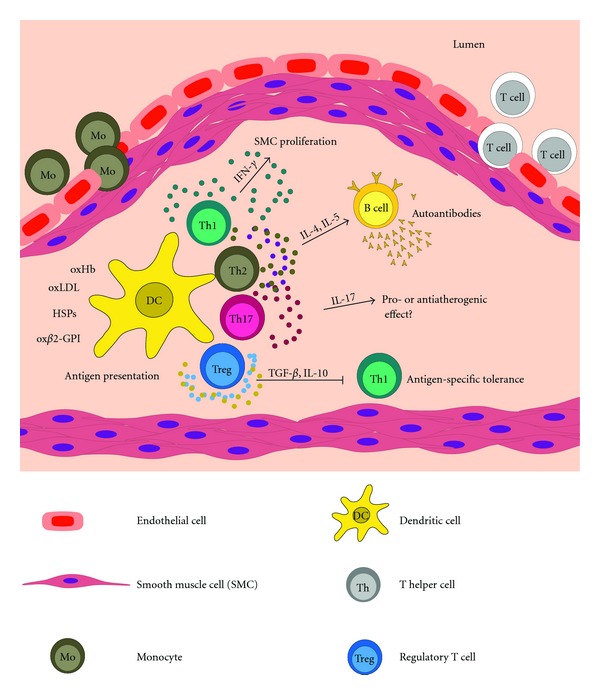
Schematic representation of T lymphocyte subset activation within atherosclerotic plaque. Different self-structures are expressed in the intima of the vessel wall and subjected to enzymatic/oxidative modifications. These modified molecules are taken up by dendritic cells and presented to CD4+ T helper cells via MHC class II molecules. Antigen presentation promotes T lymphocyte activation and secretion of different cytokines. Effector CD4+ Th1 cells produce pro-inflammatory cytokines, particularly IFN-*γ*, with proatherogenic effects. Activated Th2 lymphocytes secrete IL-4 and IL-5 which promote the differentiation of B cells in plasma cells and the production of specific antibodies. Some infiltrated T cells upon activation differentiate in Th17 lymphocytes characterized by the production of IL-17, but their role remains controversial. Regulatory T cells (Tregs) downregulate inflammation by secretion of TGF-*β* and IL-10. The pro-inflammatory mediators released by activated T cells reduce the stability of the lesion promoting plaque rupture and thrombotic events.

All these data strongly suggest that different molecules are responsible for the activation of pro-inflammatory T-cell-mediated immune responses and support the multifactorial nature of the atherosclerotic disease.  Figure 1 summarizes the activation of T lymphocyte subsets within the atherosclerotic plaque.

## 4. Conclusions

Low-grade chronic inflammation is the major pathophysiological factor contributing to the development of atherosclerosis. Chronic activation of autoimmune reactions against self-proteins modified by oxidative stress may contribute to local and systemic inflammation, thus sustaining endothelial dysfunction and plaque development in patients with cardiovascular diseases. 

Autoreactive T lymphocytes within the lesion or in the periphery can influence the atherosclerotic process by producing pro- and anti-inflammatory mediators and promoting macrophage and vascular tissue activation. Therefore, modulation of the adaptive immune responses could represent a useful approach to prevent and/or treat atherosclerotic disease. Clinical studies aimed to immunomodulate atherosclerosis are ongoing. In particular clinical trials with anti-LDL antibodies and vaccination studies with LDLs are in progress [107, 108]. A promising approach for future atherosclerosis vaccine development is to induce tolerance against atherosclerosis-associated T-cell antigens. A frequently used approach to induce tolerance is by mucosal immunization [108]. It has been demonstrated that oral administration of oxidized LDLs is associated with suppression of atherosclerosis and induction of Tregs in peripheral lymphoid tissues [109]. The same results have been obtained by the use of mucosal immunization with HSP60/65 and *β*2-GPI [110, 111]. More studies are necessary to better understand the role played by autoimmune responses in inflammatory mechanisms responsible for endothelial dysfunction to set up effective strategies of vaccination in atherosclerotic diseases.
